# Preoperative skin asepsis in bovine surgery: an outcome-blinded 3-arm randomized clinical trial under non-sterile operating room conditions

**DOI:** 10.3389/fvets.2024.1446649

**Published:** 2024-12-06

**Authors:** Emma Marchionatti, Caroline Constant, Adrian Steiner

**Affiliations:** ^1^Clinic for Ruminants, Department of Clinical Veterinary Science, Vetsuisse Faculty, University of Bern, Bern, Switzerland; ^2^Graduate School for Health Sciences (GHS), University of Bern, Bern, Switzerland; ^3^AO Research Institute Davos, Davos, Switzerland

**Keywords:** preoperative skin asepsis, RCT, skin decolonization, chlorhexidine, povidoneiodine, octenidine, surgical site infection, cattle

## Abstract

**Background:**

Surgical site infections (SSI) following clean abdominal surgery in cattle pose significant economic and welfare concerns. Preoperative skin asepsis aims to minimize microbial load over the surgical field before and throughout surgery to minimize its risk. While chlorhexidine (CHX) and povidone-iodine (PVI) are commonly used antiseptics for this purpose, our study introduces the so far unexplored use of octenidine (OCT) in veterinary surgery.

**Methods:**

We compared in a single-center, prospective, randomized, outcome-blinded, 3-arm trial the effectiveness of an OCT-based protocol to CHX- and PVI-based protocols. Cattle aged 2 years or older, needing a clean standing flank laparotomy (e.g., exploratory laparotomy, right flank omentopexy, left flank abomasopexy), were eligible. Exclusion criteria comprised skin abnormalities, recent antibiotic use, or debilitating conditions with systemic involvement or distant site infections. Patients were randomized 1:1:1 with concealed allocation using unique identifiers. Skin bacterial reduction (immediate [IRF] and delayed [DRF] reduction factors), SSI rate, and wound scores were evaluated. Wound scores were rated on a 0 to 6 scale, considering the presence and severity of discharge and swelling.

**Results:**

Out of 373 assessed cattle, 126 were included and randomized: PVI (*n* = 42), CHX (*n* = 41), OCT (*n* = 43). All protocols significantly reduced bacterial counts, with PVI exhibiting lower IRF. No significant differences were observed in DRF. The summer season and duration of surgical procedures had a negative impact on IRF and DRF in all groups, respectively. Nine of 118 patients (7.6%) with complete follow-up developed SSI. Higher wound scores were associated with development of SSI. Wound scores ≥3 at day 10 postoperatively predicted SSI with high sensitivity and specificity. Microbial flora in SSI included typical skin bacteria and opportunistic pathogens.

**Conclusion:**

All protocols met minimum FDA standards in reducing bacterial colonization. While limited by sample size and single-center design, this study confirms the OCT-based protocol as a valuable option for preoperative skin asepsis in clean abdominal bovine surgery.

## 1 Introduction

The incidence of surgical site infection (SSI) following clean abdominal surgery in cattle ranges from 4.4 to 10.5% depending on the study setting, potentially constituting a substantial economic and welfare burden (1–3). Although specific economic evaluations of SSI in bovine surgery are lacking, cows with SSI following cesarean sections have been shown to have 4.8 times higher odds of being culled (4). Moreover, SSI may adversely affect productivity, notably milk production, thereby impacting both profitability and longevity of the affected animals within the herd (5). Since most bacteria responsible for SSI following clean surgery typically originate from the patient’s own skin flora, preoperative skin asepsis aims to minimize the bioburden over the surgical field before and until the end of surgery (6). The Centers for Disease Control and Prevention (CDC) strongly recommends that patients undergo skin decontamination with an antiseptic soap before entering the operating room, and preoperative skin preparation with alcohol-based antiseptic agents (7). Although no such recommendations exist in bovine surgery, commonly used practices involve a skin decontaminating wash with a neutral or antiseptic soap followed by an aseptic final preparation (1–3). Various antiseptics can be used for this purpose, including chlorhexidine (CHX) or povidone-iodine (PVI) as aqueous or alcohol-based products. Despite evidence favoring CHX-based protocols in human surgery (8, 9), studies in bovine surgery comparing CHX- and PVI-based protocols concluded that both effectively reduce bacterial counts, with no significant difference in SSI frequency (1, 2, 10).

Concerns have been raised regarding the (independent) use of both CHX and PVI for preoperative skin asepsis. CHX has been associated with reduced susceptibility and even acquired antiseptic resistance with widespread use (11–13). Despite its traditionally credited prolonged residual effect and increased dermal tolerance compared to PVI, these characteristics may have been overemphasized (14, 15). Octenidine (OCT), available commercially as skin, mucous membrane, and wound antisepsis, as well as patient decolonization product, for over 35 years in Europe and later in Australia and Asia, offers several advantages (16–18). It is highly effective within short exposure times and in the presence of interfering substances such as blood or mucin, and it has a broad spectrum of antimicrobial activity against both Gram-positive and Gram-negative bacteria, as well as fungi, including multidrug-resistant strains (19–26). In humans, OCT is also associated with a residual effect of at least 48 h (27, 28) and exhibits good dermal tolerance (29). In contrast to CHX, OCT does not induce resistance or cross-resistance to last resort antibiotics, likely due to its non-specific mode of action, which involves purely physical interactions with the lipid membranes of microorganisms (30, 31).

However, OCT has not yet been established for preoperative skin asepsis in veterinary medicine, and data regarding its effectiveness on large animals are lacking. This randomized outcome-blinded controlled trial aims to compare an OCT-based preoperative skin asepsis protocol to the current standard of care, CHX- and PVI-based protocols, for clean abdominal bovine surgery, focusing on skin bacterial reduction and the incidence of SSI.

## 2 Materials and methods

This single-center, prospective, randomized, outcome-blinded, 3-arm parallel controlled trial was approved by the Committee for Animal Experiments Federal Food Safety and Veterinary Office of the Canton of Bern, Switzerland (BE95/20), and registered as FSVO-trial number 32691. The trial recruitment phase took place at the Clinic for Ruminants, University of Bern, Switzerland, from January 2021 to June 2023; data analysis occurred from December 2023 to March 2024. CONSORT guidelines were implemented (32).

### 2.1 Patients and inclusion criteria

Patients eligible to be included were all cattle aged 2 years or older in need of a clean standing flank laparotomy for treatment or diagnostic purposes (e.g., exploratory laparotomy, right flank omentopexy, left flank abomasopexy) and for whom owners provided written consent to participate in the clinical trial. Exclusion criteria were the presence of skin cuts/abrasions over the intended surgical field, history of skin disorders, known allergies to the used products, recent application of systemic antibiotics (<2 weeks before surgery), and debilitating conditions with systemic involvement (e.g., metritis, mastitis, pneumonia) or distant site infections (e.g., septic arthritis). Patients were randomized to one of the 3 preoperative protocols (PVI, CHX, OCT), and randomization was done by ballot draw with a 1:1:1 allocation ratio using block sizes of 9. The allocation process was concealed from the veterinarian responsible for evaluating eligibility and enrolling the patients in the study. After enrollment, they were assigned a unique 9-digit identifier created by the central admission of the Animal Hospital. This identifier was then connected to a sequential study number, ensuring the concealment of allocation from those assessing the outcomes and analyzing the data.

The following information was collected for all enrolled patients: age, sex, breed, season, type of surgery, duration of surgery, and surgeon’s experience. Immediately before surgical intervention, all enrolled patients received a systemic antibiotic (oxytetracycline 5 mg/kg IV preoperatively and continued BID for 3 days) and a non-steroidal anti-inflammatory drug (flunixine meglumine 2.2 mg/kg IV preoperatively and continued SID for 2 days) as part of the standard protocol before clean abdominal surgery used at our department. The surgical wounds were routinely closed in three layers as per standard procedure. The peritoneum, transverse, and internal oblique muscles were sutured together using a simple continuous pattern with polyglactin 910 USP 3. The external oblique muscle was also closed with a simple continuous pattern with polyglactin 910 USP 3, while the skin layer was sutured using a Ford interlocking pattern with polyamide USP 3. Each suture layer was irrigated with sterile saline solution before proceeding to the next layer.

### 2.2 Skin antisepsis protocols

All patients were brought to the operating room and placed in standing position in a surgery chute. The coat was groomed to remove any debris, and the tail was secured. The surgical field was then clipped using a battery-operated clipper with a #40 blade over 20 cm on each side of the planned skin incision. After clipping, loose hair was brushed off the patient. A locoregional flank anesthesia (Lidocaine 2%, Streuli Pharma AG) using a paravertebral block was applied following the decontaminating wash and before the final aseptic preparation. The preoperative skin protocols consisted of two 3-min contact time decontaminating wash using PVI (Betadine^®^ [7.5 mg/mL iodine], Mundipharma Medical Company), CHX (Hibiscrub^®^ [40 mg/mL chlorhexidine digluconate], CPS Cito Pharma Services GmbH) or OCT (octenisan^®^ [3 mg/mL octenidine dihydrochloride], Schülke & Mayr AG) water-based soap rinsed with tap water and dried with clean paper towels, followed by three 1-min application of PVI (Betaseptic^®^ [3.24 mg iodine, 389 mg isopropanol, 389 mg ethanol/ml], Mundipharma Medical Company), CHX (Softasept^®^ CHX uncolored [20 mg chlorhexidine digluconate, 552.7 mg isopropanol/ml], B. Braun Medical AG) or OCT (octeniderm^®^ uncolored [1 mg octenidine dihydrochloride, 300 mg propanol, 450 mg isopropanol/ml], Schülke & Mayr AG) hydroalcoholic solution according to the patient’s allocation group. The decontaminating wash was performed with a non-sterile brush used in a back-and-forth motion in the center of the clipped skin area followed by increasingly larger, concentric circles, while the hydroalcoholic solution was applied using soaked gauzes, in circular motions, beginning at the intended skin incision and moving outward toward the edges of the surgical field. After the decontaminating wash, the brush and paper towels were required to be visibly clean before proceeding to the final asepsis preparation. A trained veterinary technician or staff nurse performed both decontaminating wash and final aseptic preparation wearing non-sterile gloves. Adverse skin reactions occurring after completion of the described protocol were recorded. The skin incision (approximately 20 cm in length) was made 10 min after the end of the skin preparation to allow complete drying of the hydroalcoholic solution.

### 2.3 Microbiologic assessment and cultures

Samples for bacterial quantification were obtained using quantitative microbial culture contact plates containing a combination of neutralizing agents such as histidine, lecithin, Tween 80 (polysorbate), and sodium thiosulfate (TSA with LTHThio ICR+, Merck Millipore®, Merck KGaA) gently pressed for 5 s in the center of the surgical field immediately after hair clipping (T0), 10 min following completion of the protocol, equaling immediately prior to surgical incision (T1), and immediately after wound closure (T2) in the operation room. Directly after the surgical procedure, the contact plates were incubated at 37°C for 48 h, and a microbiologist blinded to the patient’s group recorded the number of bacterial colony-forming units (CFU). In cases of high bacterial load, i.e., if CFU were indistinguishable from each other, a count of 1,000 CFU was assigned. The immediate (IRF) and delayed (DRF) bacterial reduction factors were defined as primary outcomes and calculated as follows:


$$IRF=\left[\frac{CFU\;T0-CFU\;T1}{CFU\;T0}\right]x100$$

$$DRF=\left[\frac{CFU\;T0-CFU\;T2}{CFU\;T0}\right]x100$$

In addition, identification of individual CFU using matrix-assisted laser desorption/ionization time-of-flight (MALDI-TOF) mass spectrometry (Bruker Daltonics GmbH, Bremen, Germany) was realized for samples at T1 and T2 if ≤20 CFUs (cut-off aimed to ensure clear and distinguishable colonies for reliable testing and accurate results) were present.

### 2.4 Follow-up

Postoperatively, surgical incisions were covered with a sterile polyurethane transparent film dressing (Opsite Flexigrid, Smith & Nephew Medical Limited) for initial 3 days, with dressing changes every 24 h. The wounds were monitored daily during hospitalization by clinic veterinary staff, and after discharge, the owner, who was carefully instructed, continued to monitor the wounds until 30 days post-operatively. Photographs of the surgical incisions were taken on postoperative days 1, 2, 3, 10, and 30 and then assessed retrospectively by a large animal surgeon specialist blinded to the patient’s allocation group. Each surgical site was scored based on the presence of swelling (0 = none, 1 = difficult to detect, 2 = easy to detect, 3 = severe) and discharge (0 = absence, 1 = small quantity of dried discharge, 2 = small quantity of wet discharge, 3 = large quantity of wet discharge). The total wound score (WS; min = 0, max = 6) was computed as the sum of swelling and discharge scores for each patient for each time point (WS1, WS2, WS3, WS10, WS30) and as the maximal wound score (MWS). When present, SSI was diagnosed and classified by the attending veterinarian, unaware of the patient’s group allocation, according to the definitions outlined in the CDC guidelines as superficial, deep, or organ/space (33). When SSI occurred, whenever possible, a sample for bacteriologic analysis was taken using a microbiology swab (Transwab^®^ Gel Amies Plain, Medical Wire & Equipment), and species identification was performed using MALDI-TOF mass spectrometry. SSI rate, defined as the number of patients that developed SSI divided by the number of patients that completed the 30-day follow-up, as well as the MWS were defined as secondary outcomes.

### 2.5 Statistical analysis

We referred to data from previous studies to establish the sample size based on primary outcomes (1, 34). With a desired power of 0.80, an alpha error of 0.05, a standard deviation of 15%, and an effect size of *f* = 0.18, calculations suggested a total sample size of 291 (97 per group) to detect the critical *F*-value (*F* = 3.03). Since data on OCT use in veterinary surgery were lacking during the initial trial planning, interim analyses for sample size reassessment were scheduled and conducted at 6-month intervals, reducing the sample size to 38 patients per group.

An intention-to-treat (ITT) statistical approach was used for the primary outcomes. Thus, all randomized patients were included in the analysis. Before inferential analyses, univariate analyses were performed to assess the individual effects of covariates and independent variables on IRF and DRF. Covariates included age and surgery time, while independent random variables encompassed sex, breed, season, type of surgery, surgeon’s experience, and study group. In addition, IRF was a covariate for DRF. Two mixed-effects models, one for each main outcome of interest (IRF, DRF), were then used for the main analysis, incorporating covariates and independent variables identified in the univariate analyses. These models addressed potential confounding factors by considering both within-group and between-group variability, with significance levels adjusted using the Bonferroni correction method for multiple analyses.

A per-protocol analysis was conducted for the secondary outcomes, including all patients who completed the 30-day follow-up period. A χ^2^ test was used to compare SSI rates among the three groups. Univariate analyses were performed to assess the effects of covariates and independent variables on SSI and MWS. Covariates included age, time of surgery, IRF, and DRF, while independent random variables included sex, type of surgery, surgeon’s experience, and study group. In addition, MWS was used as a covariate for SSI. Similarly, two mixed-effects models, one for each main outcome of interest (MWS, SSI), were used for the main analysis, adjusting for significant variables identified in the univariate analyses and employing the Bonferroni correction for multiple comparisons.

To predict SSI, a mixed-effect model for repeated measures was initially used to evaluate the impact of the different wound scores (WS1, WS2, WS3, WS10, WS30, MWS) on SSI, with Sidak’s correction for multiple comparison tests applied. Subsequently, statistically significant results were further investigated using receiver-operating characteristic (ROC) curves to identify the most predictive factor for the occurrence of SSIs, with sensitivity (Se) and specificity (Sp) assessed using the Youden’s index and the area under the ROC curve (AUC).

Data analysis was performed using SPSS® version 29.0 (IBM, Armonk, New York, United States) and Prism version 8.4.3 (GraphPad Software, San Diego, California, United States).

## 3 Results

**Figure 1:**
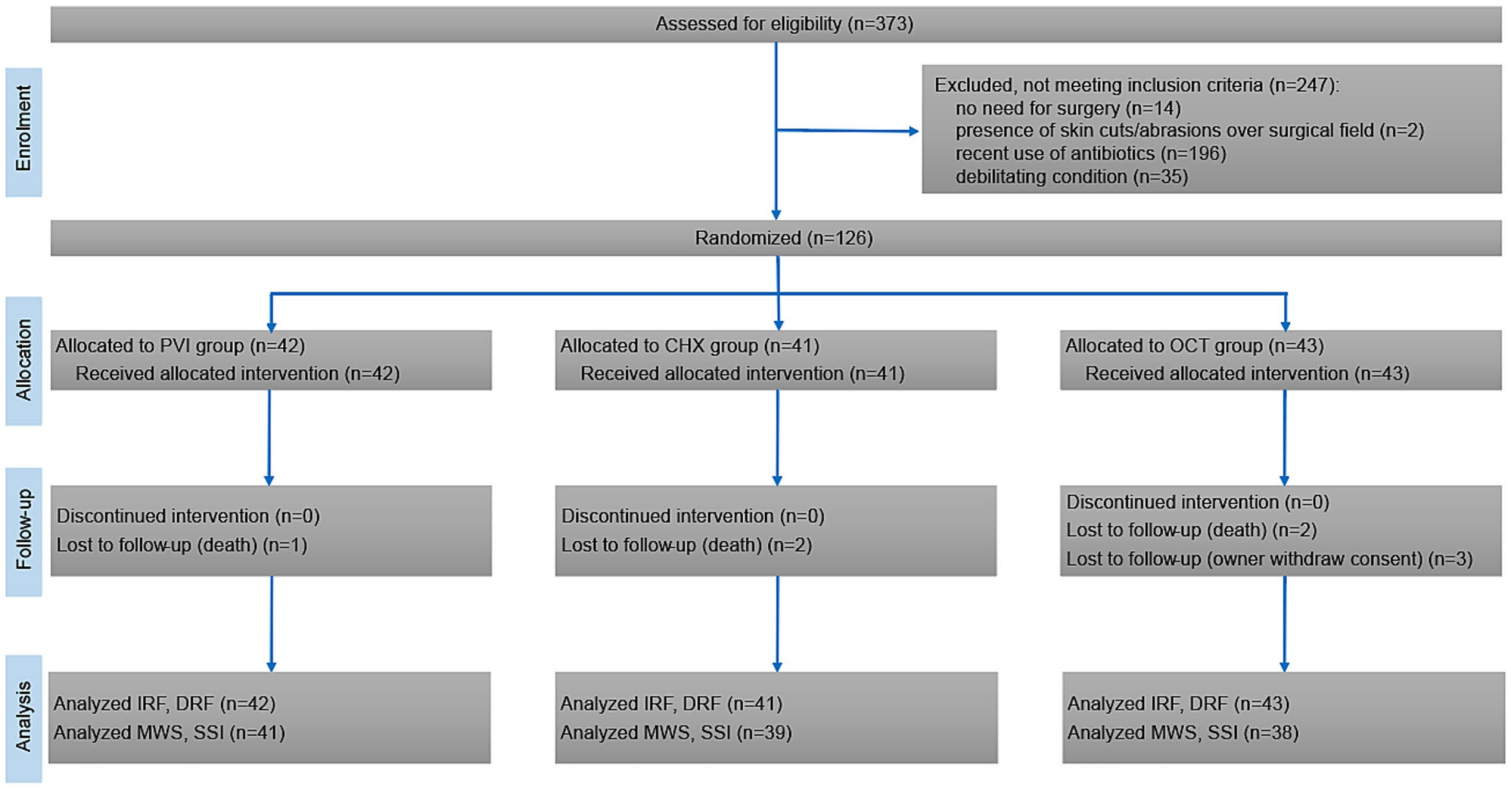
CONSORT flow diagram showing inclusion, randomization, and participation of patients throughout the study.

Three hundred seventy-three cattle were assessed for eligibility between January 1st, 2021 and June 30th, 2023. Out of these, 247 were excluded due to one or more exclusion criteria, with recent use of antibiotics being the most frequent (*n* = 196). This resulted in a total of 126 patients being randomized in the three study groups: PVI (*n* = 42), CHX (*n* = 41), and OCT (*n* = 43). All randomized patients received the intended intervention, and no deviations from the study protocol were reported. During the 30-day follow-up period, 3 owners withdrew their consent for their animals to participate in the follow-up, and 5 animals died (slaughter for low milk production, *n* = 3; sudden death due to abomasal ulcers, *n* = 2). Figure 1 documents the number of patients through the trial. The baseline information of the patients according to their group is shown in Table 1. No significant adverse events were reported following antiseptic application, except for one patient in the PVI group who experienced mild dermatitis characterized by diffuse erythema and mild skin edema. This condition was resolved within 24 h without necessitating further interventions.

**Table 1:** Baseline information of patients in the three study groups.

	Total (*n* = 126)	PVI (*n* = 42)	CHX (*n* = 41)	OCT (*n* = 43)
Age (years)				
<3	46	13 (31.0)	17 (41.4)	16 (37.2)
3–5	50	21 (50.0)	11 (26.9)	19 (44.2)
>5	30	8 (19.0)	13 (31.7)	8 (18.6)
Sex Ratio (M:F)	2:126	0:42	1:41	1:43
Breed				
Red Holstein	44	17 (40.5)	11 (26.8)	16 (37.2)
Holstein Fresian	63	19 (45.2)	23 (56.1)	21 (48.8)
Swiss Fleckvieh	16	6 (14.3)	6 (14.6)	4 (9.3)
Others	3	0 (0.0)	1 (2.5)	2 (4.7)
Season				
Spring	54	18 (42.9)	14 (34.1)	22 (51.2)
Summer	13	3 (7.1)	5 (12.2)	5 (11.6)
Autumn	20	7 (16.7)	8 (19.6)	5 (11.6)
Winter	39	14 (33.3)	14 (34.1)	11 (25.6)
Type of Surgery				
Right Flank Omentopexy	112	38 (90.5)	37 (90.2)	37 (86.0)
Explorative Laparotomy	14	4 (9.5)	4 (9.8)	6 (14.0)
Duration of Surgery (min)				
<60	8	2 (4.8)	2 (4.8)	4 (9.3)
60–120	111	36 (85.7)	39 (95.2)	36 (83.7)
>120	7	4 (9.5)	0 (0.0)	3 (7.0)
Surgeon’s Experience (years)				
<2	20	7 (16.7)	5 (12.2)	8 (18.6)
2–5	90	32 (76.2)	28 (68.2)	30 (69.8)
>5	16	3 (7.1)	8 (19.6)	5 (11.6)

Number of patients are reported for each variable; values in parenthesis represent percentages. PVI, povidone iodine; CHX, chlorhexidine; OCT, octenidine.

**Figure 2:**
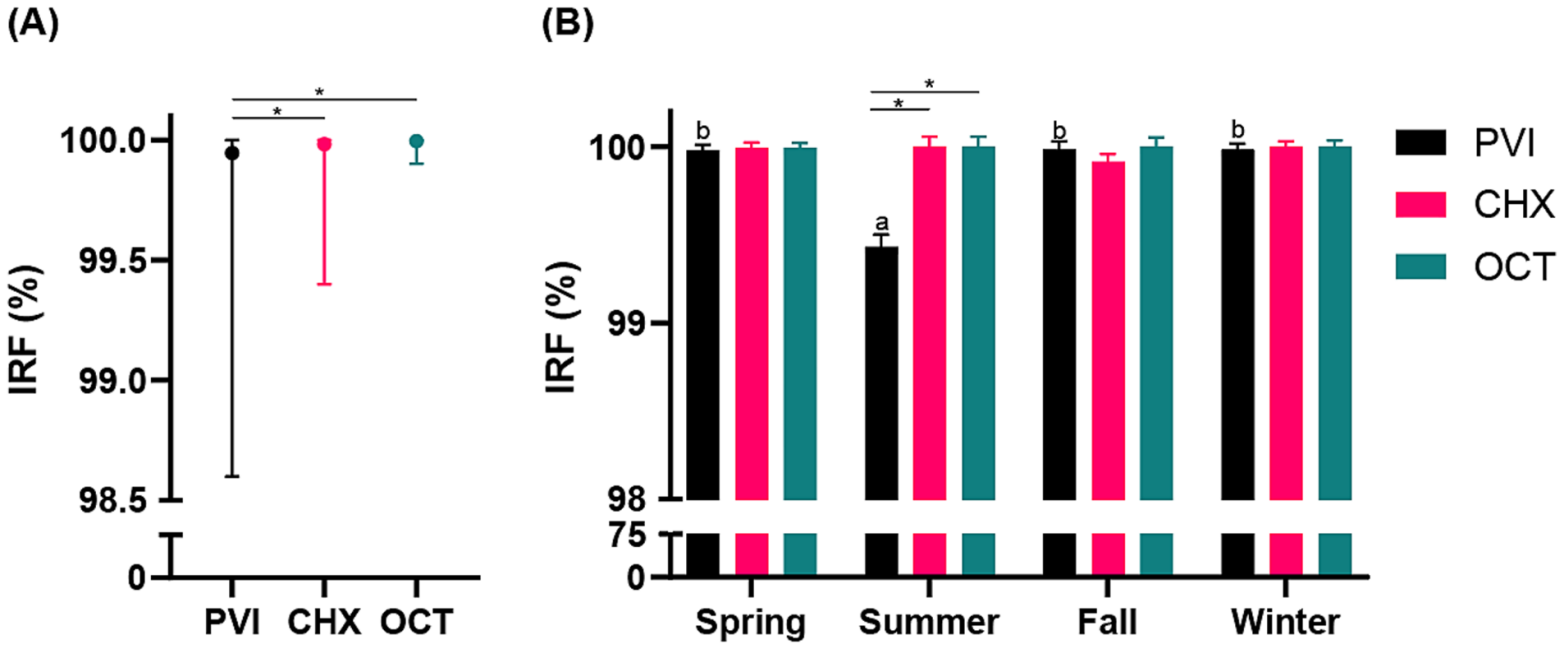
(A) Line graph illustrating the mean (●) and range (bars) for the comparison among the three study groups (PVI, povidone iodine; CHX, chlorhexidine; OCT, octenidine) regarding the immediate reduction factors (IRF; %). (B) Histogram showing the interaction between study group (PVI, CHX, OCT) and season (spring, summer, fall, winter) concerning the IRF (%). Statistically significant differences are indicated by a superscript asterisk (*) and letters (a, b).

As depicted in Figure 2, the three investigated protocols resulted in a substantial decrease in CFUs preoperatively compared to baseline (IRF: 99.94% ± 0.22% [range 98.6 to 100%] PVI, 99.98% ± 0.09% [range 99.4 to 100%] CHX, 99.99% ± 0.02% [range 99.9 to 100%] OCT), corresponding to a reduction of at least a 3 log_10_. This reduction was sustained postoperatively (DRF: 97.02% ± 3.62% [range 86.7 to 99.6%] PVI, 98.16% ± 3.46% [range 82.1 to 100%] CHX, 96.98% ± 4.80% [range 78.9 to 100%] OCT). Univariate analysis indicated significant variations in IRF associated with seasonal changes (*p* = 0.016). Consequently, season was incorporated in the mixed-effects model to assess the impact of the study group on IRF. Results showed that both season (*p* < 0.001) and study group (*p* < 0.001) significantly affected IRF. Specifically, the PVI group exhibited lower IRF compared to both CHX (*p* < 0.01) and OCT (*p* < 0.01) groups (Figure 2A), with the summer season associated with the lowest IRF (*p* < 0.01). Moreover, the model revealed a significant interaction between the study group and season (*p* < 0.001; Figure 2B). Univariate analysis also revealed significant variations in DRF linked to the duration of surgery (*p* = 0.003). Thus, surgery time was included in the mixed-effects model to assess the effect of the study group on DRF. The analysis demonstrated that DRF did not statistically differ between the groups (*p* = 0.588), but duration of surgical procedure significantly impacted DRF (*p* = 0.004) regardless of the study group (Supplementary Figure S1). Surgery duration ranged between 30 and 175 min (85 min ± 21 min).

The overall SSI rate was 7.6% (*n* = 9 out of 118 patients with 30-day follow-up). Superficial SSI developed in 7 patients (PVI *n* = 2; CHX *n* = 2; OCT *n* = 3), whereas 2 deep SSI were reported in the PVI group; organ-space SSI did not occur in any patients. There was no difference in the overall SSI rate between the study groups [9.8% (4 of 41) with PVI; 5.3% (2 of 38) with CHX; 7.7% (3 of 39) with OCT; *p* = 0.72]. None of the animals where surgeries lasted over 2 h developed SSI. Univariate analysis indicated that age (*p* = 0.041) and breed (*p* = 0.004) were significant predictors of MWS. Therefore, these variables were included in the mixed-effects model evaluating the effect of the study group on the MWS and were confirmed to have a significant influence on the MWS outcome (*p* = 0.004) (Supplementary Figure S2). The univariate analysis found that both breed (*p* = 0.019) and MWS (*p* < 0.001) were associated with SSI. However, in the mixed-effects model, only MWS remained a significant predictor of SSI risk (*p* = 0.009; Supplementary Figure S3).

**Figure 3:**
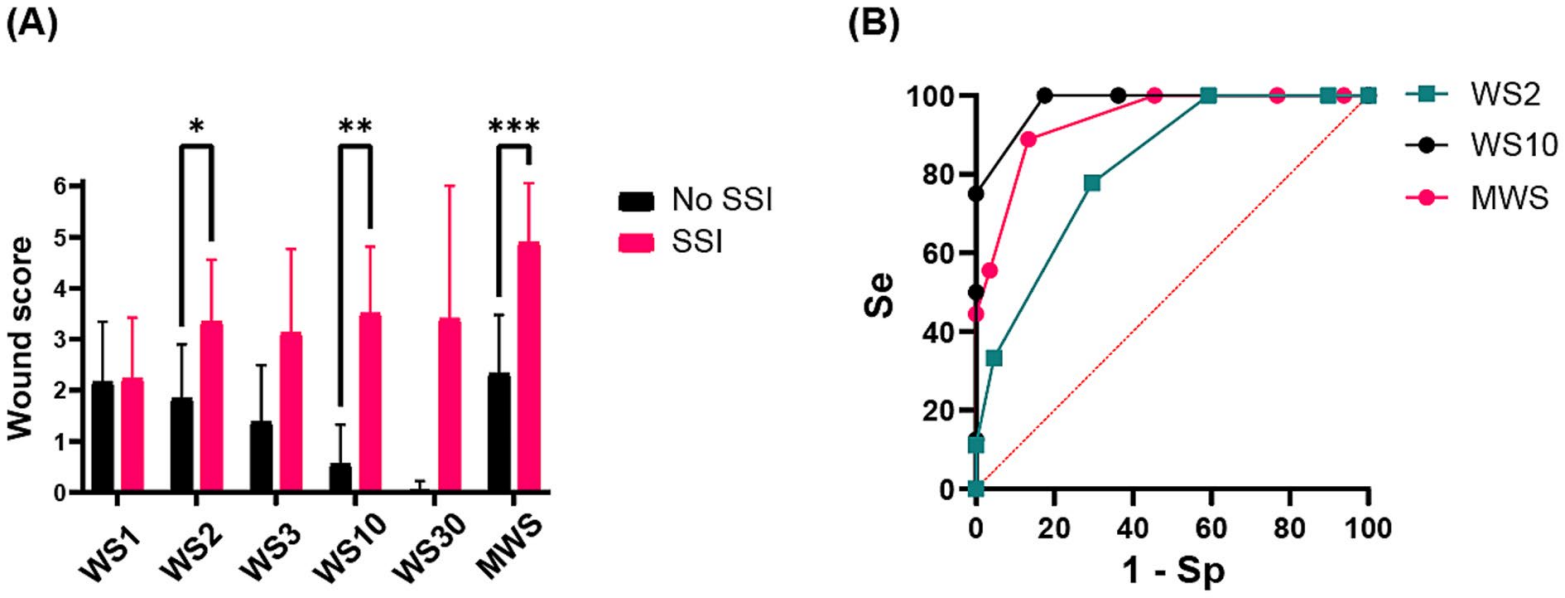
(A) Histogram showing the relationship between mean ± standard deviation (SD) wound scores (WS1 = wound score at day 1, WS2 = wound score at day 2, WS3 = wound score at day 3, WS10 = wound score at day 10, WS30 = wound score at day 30, MWS, maximal wound score) and surgical site infection (SSI) development. Statistically significant differences are indicated by superscript asterisks (*). (B) Receiver operating characteristic (ROC) curve sensitivity/specificity (Se/Sp) analysis of the predictor values of WS2, WS10 and MWS.

Incidence of SSI was significantly associated with the wound scores (*p* < 0.001) and especially with WS2 (*p* = 0.036), WS10 (*p* = 0.002), and MWS (*p* = 0.001), respectively. The predictor value of WS10 (AUC: 0.9787, *p* < 0.001) was superior to the others (Figure 3A). The ROC curve and Se/Sp analyses revealed that a wound score of ≥3 at day 10 used as a cut-off point yields a sensitivity of 75% and specificity of 100% for the prediction of SSI (Figure 3B).

The bacteria detected at T1 and T2 primarily belonged to the *Staphylococcaceae*, *Corynebacteriaceae*, *Aerococcaceae*, and *Bacillaceae* families, bacteria commonly found on the normal skin or in the environment. These were identified in 3.8, 0.8, 2.4, and 3.8% of T1 positive cultures (*n* = 11) and 57.9, 17.5, 21.5, and 4.0% of T2 positive cultures (*n* = 106), respectively. Bacteria isolated from 5 positive cultures issued from SSI comprised *Trueperella pyogenes* (*n* = 4), *Corynebacterium jeikeium* (*n* = 1), *Escherichia coli* (*n* = 1), *Fusobacterium necrophorum* (*n* = 1), and *Enterococcus faecalis* (*n* = 1); when *T. pyogenes* was isolated, it was commonly in poly bacterial culture (*n* = 3).

## 4 Discussion

Preoperative skin asepsis aims to reduce the microbial load over the surgical field to a minimum before and until the end of surgery, with the ultimate goal of supporting SSI prevention. According to the Food and Drug Administration (FDA) criteria, the bioburden reduction within 10 min should be at least 2 log_10_ for dry sites and 3 log_10_ for moist sites, without a return to baseline for at least 6 h post-application (35). All three investigated protocols in this RCT met these minimum standards for microbial reduction, by reducing skin microflora by at least 3 log_10_ before surgery, and although we did not assess microbial counts 6 h post application, none of the protocols exhibited a return to baseline bioburden by the end of surgery. Further data analysis revealed that the PVI group exhibited significantly lower IRF than CHX and OCT groups, while no difference was observed in DRF between the three protocols. The observed SSI rate in our study was 7.6%, consistent with findings from previous research (1–3), but significant differences were not observed among the three protocols.

While antiseptic agents play a crucial role in preoperative skin preparation, it is essential to acknowledge that factors beyond the choice of an antiseptic product, like duration of surgery and the hospital environment, significantly impact bacterial load and, consequently, SSI occurrence. In teaching hospitals, students’ involvement and inexperienced operators may prolong surgical time, thus increasing the risk of SSI. Preoperative skin aseptic preparation reduces the amount of pathogens on the skin, but complete skin sterilization is not possible, leading to gradual recolonization already during surgery. In the setting of the present study, prolonged surgical intervention correlated with higher postoperative microbial load at the surgical site regardless of the investigated protocol. However, while DRF did not correlate directly with SSI, the MWS did, and wound scores ≥3 on day 10 could accurately predict SSI development. Moreover, the surgical procedures of the present study were performed in a non-sterile surgery room on conscious, standing cattle able to move and defecate throughout the surgical procedure, contributing to environmental contamination and increased risk of wound contamination during the operation. The impact of the season should not be neglected either, as warm and humid conditions pose favorable conditions for bacterial growth, potentially increasing the risk of SSI (36). Although in our study summer months were associated with higher microbial loads prior to surgery, this season did neither impact the bioburden postoperatively nor the frequency of SSI. Still, this clinical trial was not powered to detect differences in SSIs between the 3 groups or seasons of the year. Noteworthy, both colorless skin antiseptic products used in the CHX and OCT study arms did not appear to have a negative impact on the performance of the preoperative skin preparation. In fact, according to IRF data, preoperative skin preparation with these products was significantly better compared to the colored PVI product. In human surgery, the use of colorless skin antiseptics has been associated with decreased skin coverage compared to colored preparations (37). However, this does not seem to be an issue in veterinary surgery. Nonetheless, further research is warranted to explore the potential impact of uncolored antiseptics on the risk of SSI in veterinary settings.

We performed digital photography and standardized wound scoring at specific time points for follow-up and to anticipate patients at risk of developing SSI. This offers an objective tool to monitor wound healing parameters like swelling and the presence of exudate and is therefore widely used in human medicine as well (38). Moreover, digital images can be easily shared electronically, enabling remote consultations, and obviating the need for veterinarians to visit the animal at the farm. This not only could save time but also reduce costs associated with on-site visits. Accurately predicting SSI occurrence is crucial for proactive surgical complication surveillance, facilitating timely interventions and averting more serious outcomes. While promising sensitivity and specificity for SSI prediction were observed at the 10-day wound scoring, the effective integration of remote postoperative assessment via photography may necessitate further refinement in veterinary practice. A trial-specific photography protocol to ensure standardization of procedures and image quality should be developed, with particular attention to lighting, background, exposure setting, use of flash, distance from the wound and angles.

As expected, the microorganisms isolated from the skin consisted mainly of bacteria, typical for the natural flora on bovine skin (39); those isolated from infected wounds included opportunistic pathogens commonly found on the skin and/or in the gastrointestinal tract, such as the rumen and intestine. Although flank omentopexy procedures for treating left or right abomasal displacement are classified as clean surgical procedures, we hypothesize that opportunistic bacteria may have reached the incision site through the needle puncture performed for gas evacuation from the abomasum. Additionally, the highly contaminated environment where standing surgery is performed in cattle poses another probable source of these pathogens.

Several factors beyond pure antimicrobial efficacy must be considered when selecting a preoperative skin aseptic protocol. Potential side effects of antiseptics should be carefully evaluated. Although it was not the primary focus of this investigation, skin reactions in enrolled patients were monitored, as it is known that certain antiseptics can induce skin irritation or allergic reactions. In humans, allergic contact dermatitis is more prevalent for CHX than PVI or OCT (40). However, data on cattle skin are limited. No skin reactions were noted following antisepsis of the paralumbar fossa in cattle when comparing CHX and alcohol in a recent experimental study (41). Nonetheless, low occurrence of dermatitis was observed in a previous study, but without differences between CHX and PVI (2). Our results on a limited number of patients showed no reactions to used antiseptics, with only one case of mild dermatitis occurring in the PVI group. Also, the emergence of reduced antiseptic susceptibility is a growing concern and should be accounted for in the choice of products. While no reports of increased tolerance neither to PVI nor OCT have been documented despite its extensive clinical use over many decades, recent studies prove CHX’s potential not only to promote reduced susceptibility among pathogens (42, 43), but also to induce cross-resistance to antibiotics in Gram-negative species (28). Cost considerations play a significant role, especially for livestock. The overall costs of the preoperative skin preparation protocols in our study were 26.5 CHF (30 USD), 35 CHF (39.5 USD), and 25.5 CHF (29 USD) for the PVI, CHX, and OCT, respectively. Given comparable efficacy, the favorable cost/benefit option in cattle was identified using OCT in this study.

Although being a controlled randomized trial, our study is not without limitations. Firstly, there is the potential for an inadequate sample size to discern differences between groups. Despite meticulous considerations in sample size determination and periodic reevaluations during interim analyses, we cannot rule out the possibility that the lack of significance in specific parameters might have resulted from a type 2 error. Additionally, the study was designed and powered to detect differences in the capability of the antiseptic regimes to reduce bacterial contamination prior to surgery rather than in the SSI rate. Moreover, the impact of systemic antibiotics on the skin microbiome represents a limitation of this study. While research has shown that systemic antibiotic therapy does not significantly alter the healthy human skin microbiome (44), no direct data is available for bovine medicine. It is reasonable to assume that the effects of systemic antibiotics, such as the oxytetracycline used in this study, on the skin microbiome of cattle would be similarly minimal. However, further research is needed to confirm whether these findings in humans are applicable to bovine species. Nonetheless, since all animals in this study received the same systemic antibiotic therapy, any potential influence on bacterial populations and CFU counts would be uniform across all study groups, minimizing its impact on between-group comparisons. Finally, this study was conducted at a single center, potentially limiting the findings’ generalizability to other patient demographics or geographic locations. However, adherence to the study protocol and the investigators’ blinding helped reduce the risks of performance or detection bias, thus strengthening the validity of our results.

In conclusion, all protocols effectively reduced bacterial colonization to minimize the risk of SSIs, confirming OCT as a valuable option to PVI or CHX in clean abdominal bovine surgery.

## Data Availability

The datasets presented in this article are available from the corresponding author on reasonable request. Request to access the dataset should be directed to Emma Marchionatti, emma.marchionatti@unibe.ch.
