# Supplementary material for: Preoperative skin asepsis in bovine surgery: an outcome-blinded 3-arm randomized clinical trial under non-sterile operating room conditions
Source: Front Vet Sci. 2024 Dec 6;11:1446649. doi: 10.3389/fvets.2024.1446649 (PMC11660802; doi:10.3389/fvets.2024.1446649)
Supplement: Supplementary file 1 [file Image_1.pdf]

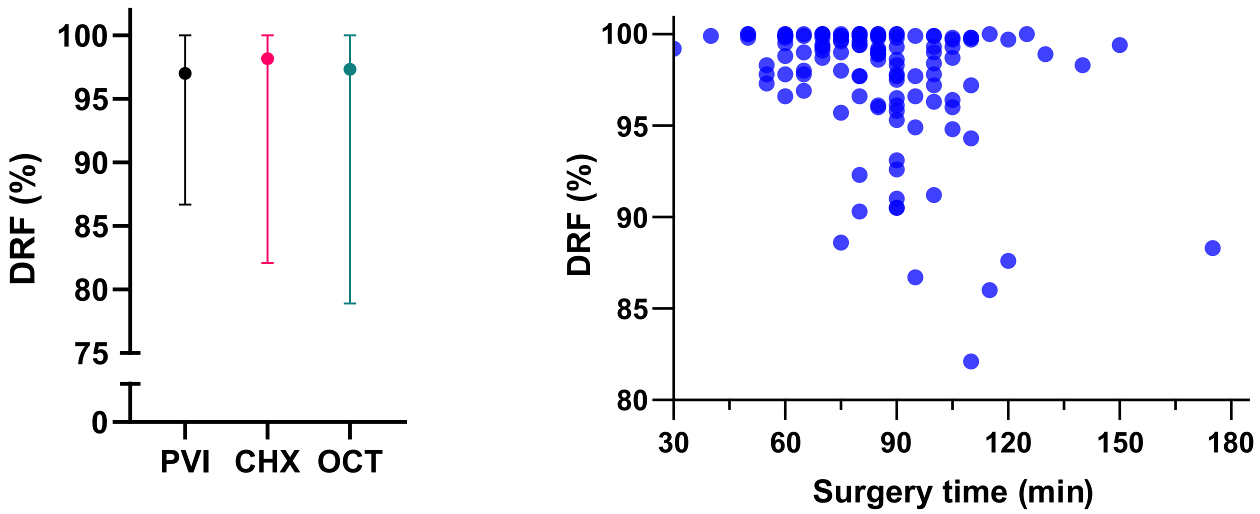

Figure S1. (A). Line graph illustrating the mean (●) and range (bars) for the comparison among the three study groups (PVI = povidone iodine, CHX = chlorhexidine, OCT = octenidine) regarding the delayed reduction factors (DRF) (%). (B) Scatterplot showing the relationship between surgery time (min) and delayed reduction factors (DRF) (%).
