# Supplementary material for: Preoperative skin asepsis in bovine surgery: an outcome-blinded 3-arm randomized clinical trial under non-sterile operating room conditions
Source: Front Vet Sci. 2024 Dec 6;11:1446649. doi: 10.3389/fvets.2024.1446649 (PMC11660802; doi:10.3389/fvets.2024.1446649)
Supplement: Supplementary file 2 [file Image_2.pdf]

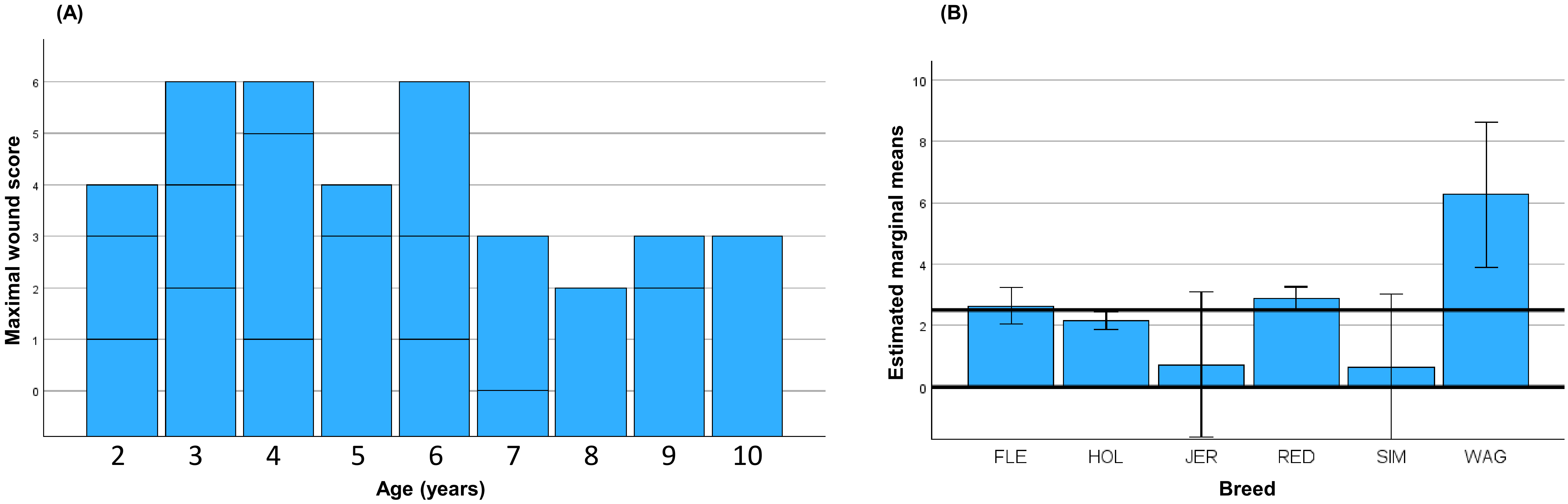

Figure S2. (A) distribution of maximal wound score by age. (B) Estimated marginal means of maximal wound score by breed; bold line corresponds to the observed grand mean and error bars to 95% CI.

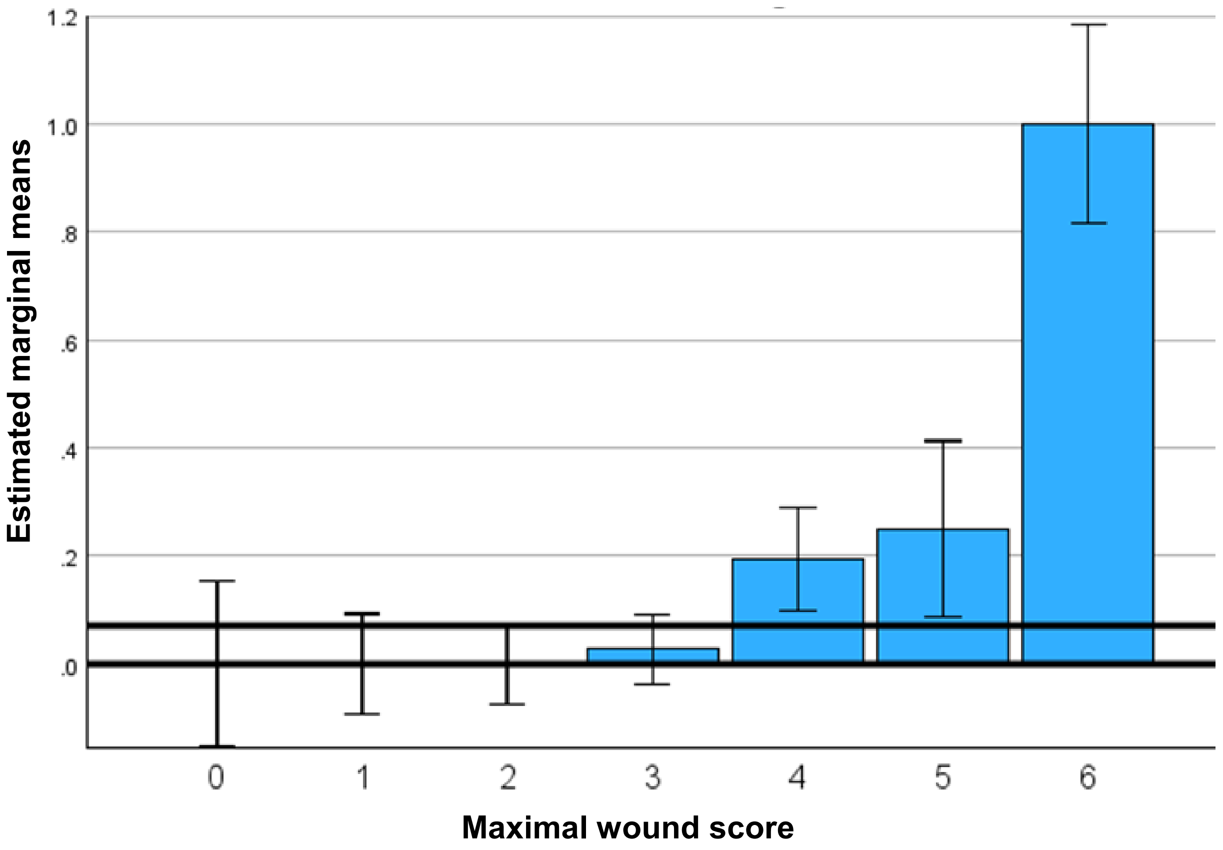

Figure S3. Estimated marginal means of SSI by maximal wound score; bold line corresponds to the observed grand mean and error bars to 95% CI.
